# Biofilm and the effect of sonication in a chronic *Staphylococcus epidermidis* orthopedic in vivo implant infection model

**DOI:** 10.1186/s13018-024-05309-3

**Published:** 2024-12-04

**Authors:** Erik Thorvaldsen Sandbakken, Erling Høyer, Eivind Witsø, Caroline Krogh Søgaard, Alberto Díez-Sánchez, Linh Hoang, Tina Strømdal Wik, Kåre Bergh

**Affiliations:** 1 Department of Orthopedic Surgery, St. Olavs Hospital, Olav Kyrres Gate 13, 7030 Trondheim, Norway; 2 Departement of Medical Microbiology, St. Olavs Hospital, Erling Skjalgsons Gate 1, 7030 Trondheim, Norway; 3 Cellular and Molecular Imaging Core Facility (CMIC), NTNU, Olav Kyrres Gate 10, 7030 Trondheim, Norway; 4 Department of Clinical and Molecular Medicine, NTNU, Erling Skjalgsons Gate 1, 7030 Trondheim, Norway; 5 Present Address: Department of Neuromedicine and Movement Science, NTNU, Olav Kyrres Gate 13, 7030 Trondheim, Norway

**Keywords:** Biofilm, Animal model, Implant infection model, *Staphylococcus epidermidis*, Sonication, PCR, Scanning electron microscopy, SEM, Epifluorescence microscopy

## Abstract

**Background:**

In diagnosing chronic orthopedic implant infections culture of sonicate represents a supplement to tissue cultures. However, the extent to which biofilm forms on implant surfaces and the degree of dislodgement of bacteria by sonication remains unclear. In this in vivo study using a low bacterial inoculum, we aimed to determine whether a variable effect of sonication could be observed in a standardized in vivo model.

**Materials and Methods:**

Seven Wistar rats underwent surgery with intramuscular implantation of two bone xenograft implants, each containing two steel plates. The grafts were inoculated with approximately 500 colony forming units (CFU) of *Staphylococcus epidermidis* ATCC 35984. After 20 days the rats were sacrificed, and the steel plates were removed from the bone grafts. Epifluorescence microscopy and scanning electron microscopy (SEM) were used to visualize biofilm formation and dislodgement on the plate surfaces. In addition to cultures of sonicate, a quantitative *S. epidermidis* specific PCR was developed for enumeration of bacteria.

**Results:**

A chronic, low-grade implant infection was successfully established, with all animals remaining in good health. All infected bone graft implants yielded abundant growth of *S. epidermidis*, with a median of 3.25 (1.6–4.6) × 10⁷ CFU per/graft. We were unable to distinguish infected plates from negative controls using epifluorescence microscopy. On infected plates small colonies of staphylococci were identified by SEM. The number of bacteria detected in the sonicate was low with 500 (100–2400) CFU/plate and 475 (140–1821) copies/plate by qPCR. The difference in area covered by fluorescent material before and after sonication was 10.1 (5.7–12.3) %, *p* = 0.018.

**Conclusion:**

Despite the pronounced infection in the surrounding tissue, only few bacteria were detected on the surface of the steel implants. This is evident from the minimal findings by SEM before sonication, as well as the very low CFU counts and DNA copies in the sonicate. Sonication did not show variable effectiveness, indicating it is a valuable addition to, but not a replacement for biopsy cultures in cases of implant-associated infections with low-virulence microorganisms.

## Introduction

Prosthetic joint surgery is a tremendous success story in modern medicine. Prosthetic joint infection (PJI), however, is a devastating complication afflicting approximately 1–2% of patients after total hip arthroplasty [1]. Differentiating aseptic versus septic loosening may be difficult but is of pivotal importance for a correct therapeutic regimen. Culture of tissue samples with subsequent antibiotic susceptibility testing has been a cornerstone for choosing correct antibiotic therapy. There is a general agreement that the sensitivity of culture is suboptimal and may be in the range 69–95%, explained by the biofilm mode of growth of bacteria on the prosthetic surface [2, 3].

Sonication, used to improve culture sensitivity by dislodging bacteria embedded in biofilms, is currently recognized as a diagnostic criterion in The European Bone and Joint Infection Society (EBJIS) guidelines but not in the The Muskuloskeletal Infection Society (MSIS) guidelines [4, 5]. This illustrates the ongoing debate whether culture of sonicate fluid is superior to culture of tissue samples. Sonication has long been regarded as a superior method [6–9].

However, the implementation of rigorous routines for harvesting tissue samples appears to increase the sensitivity of culture of tissue samples [2, 3, 5, 10].

Earlier work by our group shows that *Staphylococcus epidermidis* biofilm growing on steel yields less colony forming units (CFU) than other common bacteria in PJI’s [11]. This gave reason to speculate that sonication failed to dislodge biofilm embedded *S. epidermidis* or that biofilm was poorly developed. Bacteria growing in a biofilm phenotype can also fail to produce colonies when transferred to agar plates [12] and inaccuracy of bacterial count could be due to aggregate formation [13]. Recently, an in vitro study has shown a highly variable effect of sonication under the conditions of a uniformly distributed biofilm [14].

Relevant in vivo models mimicking chronic biofilm implant infections and how to detect bacteria consistently are scarce. Many in vivo biofilm models establish biofilm on the implant prior to implantation [15–17]. Clinical infections, however, mostly originate from a minute number of bacteria contaminating the wound during surgery [18]. To imitate this condition a chronic implant infection model utilizing a low inoculum *Staphylococcus epidermidis* during implantation has showed the establishment of a chronic biofilm infection on steel plates [19].

Previous in vivo studies have successfully employed fluorescence microscopy to visualize biofilm on the surface of implants [15]. However, in vivo biofilm is not easily interpreted as both autofluorescence, and artefacts must be considered [20]. Scanning electron microscopy (SEM) offers higher magnification and resolution with the possibility of morphological identification of single bacteria, but also makes it more difficult to estimate the extent of an in vivo biofilm. Nevertheless, it is shown that quantification of biofilm covered area by SEM can verify biofilm maturation up to 28 days after insertion of a flat implant [17].

The abovementioned observations regarding biofilm development on a steel surface and the impact of sonication raise the question if the variable effect of sonication seen in vitro also applies to in vivo biofilm. This could in part explain why cultures of sonicate in some studies may appear less sensitive than optimized tissue cultures. Studies of biofilm require quantitative methods, and we are not aware of any in vivo studies investigating the reliability of sonication to measure dislodged biofilm embedded *S. epidermidis.* We included real-time quantitative PCR (qPCR) to determine the number of bacteria without the risk of underestimates associated with counting of CFU.

In the present study we pursue our previous work on an in vivo model that mimics a chronic orthopedic low inoculum implant infection. Steel plates were seeded with *S. epidermidis *in vivo, and after 20 days subjected to sonication. In the sonicate we measured the number of live bacteria (CFU/mL) and the total bacterial load by real-time qPCR (copies/mL). Using two quantitative bacteriological methods along with visualization of bacteria on steel plates, we aimed to determine whether a variable effect of sonication could be observed in a standardized in vivo model.

### Methods

#### Methodological overview

**Fig. 1:**
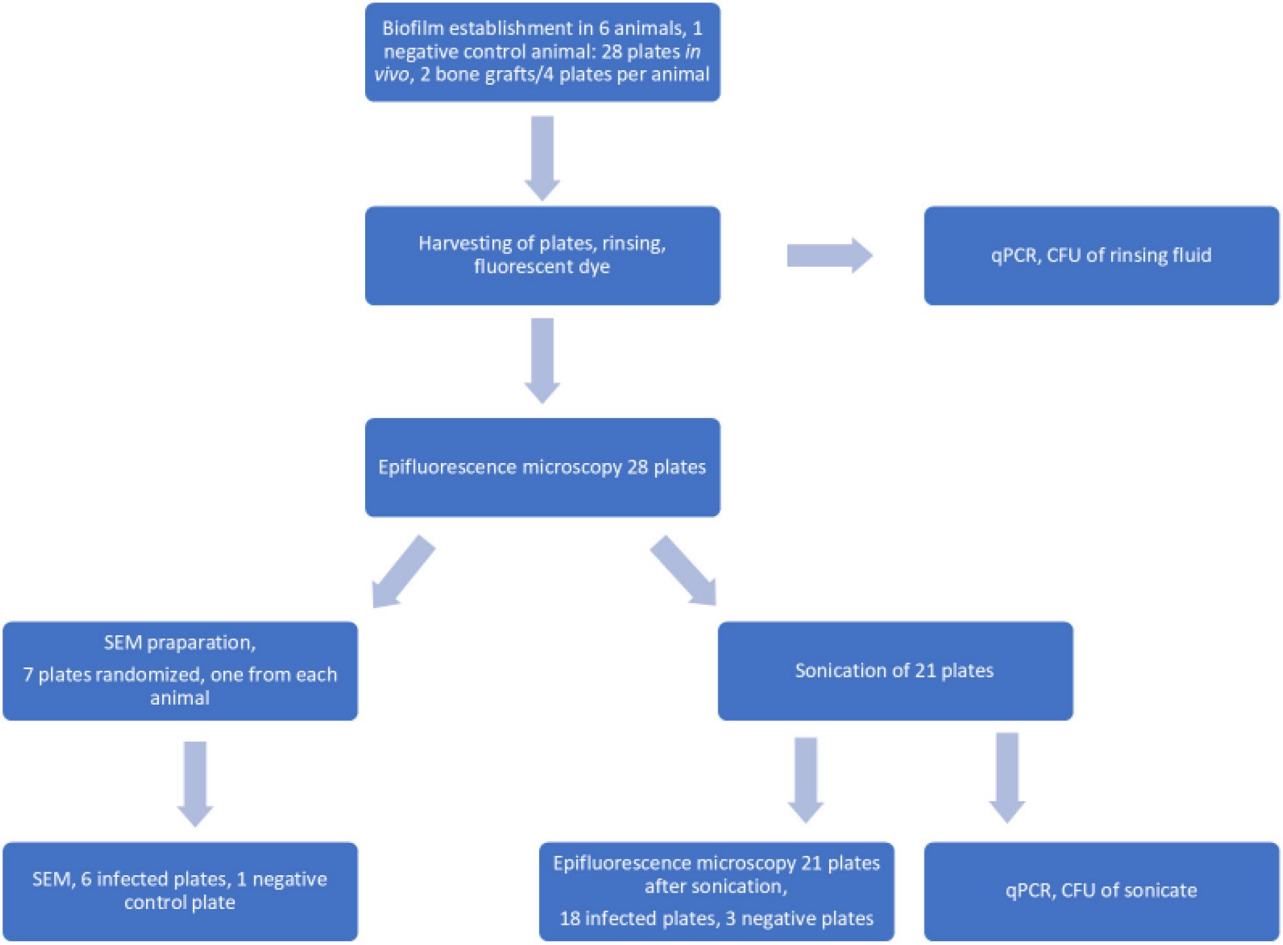
A flowchart of the experimental design outlines the steps taken to study in vivo biofilm formation. A total of 24 inoculated steel plates, along with 4 additional negative control plates, were implanted into corticocancellous bone grafts from lamb costae and placed in a subfascial cavity. After 20 days, the plates were harvested. All 28 plates, including the controls, were first analyzed using epifluorescence microscopy before sonication. Six of the 24 inoculated plates, along with one of the four negative controls, were then processed with SEM to confirm biofilm formation and to ensure that the SEM preparation preserved the biofilm structure. The remaining 18 inoculated plates and 3 negative controls underwent sonication, followed by visualization with epifluorescence microscopy and SEM. The rinsing fluid and sonicate were quantified using both culture and qPCR methods

The experimental design is shown in Fig. 1.

**Fig. 2:**
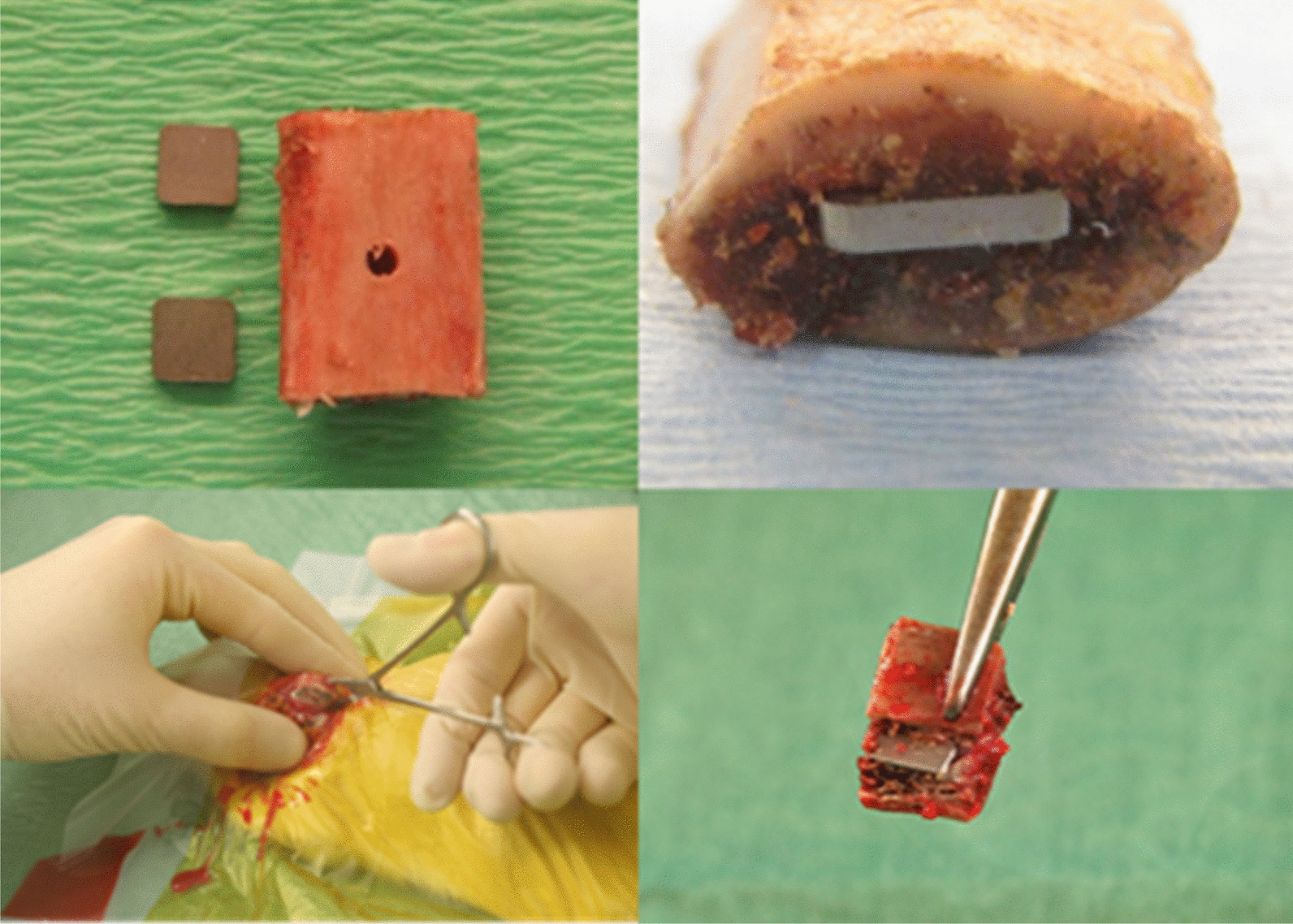
Preparation of bone grafts. (The figure was earlier published by Witsø et al. [19], https://www.ncbi.nlm.nih.gov/pmc/articles/PMC6975053/, licensed under CC BY 4.0, https://creativecommons.org/, and modified with two additional pictures showing the surgical procedure). A 15 mm-long pieces of corticocancellous bone grafts are cut from the midsection of lamb ribs. A groove, matching the size of the steel plates, is made at each end of the bone graft using a mandrel. A 2 mm hole is then drilled into the bone marrow at the center of the graft. After separately sterilizing both the bone grafts and steel plates, the plates are gently pressed into the pre-made grooves under sterile conditions. Immediately before implantation, the plates are inoculated with a bacterial suspension containing 1000 CFU in 10 µL

The animals were operated with intramuscular implantation of two bone graft implants each containing two steel plates based on previous studies [15, 19]. An intended inoculum of approximately 1000 CFU of *Staphylococcus epidermidis* (ATCC 35984) was peroperatively introduced into the bone graft implant. After 20 days the rats were sacrificed, and the steel plates were explanted from the bone grafts and further studied (Fig. 2).

We considered the infection a chronic orthopedic implant infection if *S. epidermidis* was cultured from the steel plates or the bone grafts after 20 days.

To address the issue whether colony counting is representative of the bacterial load present we developed a *S. epidermidis* quantitative real-time PCR. Measurements of CFU and qPCR from the sonicate were recorded.

Biofilm would be identified and attempted quantified using epifluorescence microscopy before and after sonication. Earlier described methods for in vitro biofilm quantification were adapted to visualize in vivo biofilm [14, 21] and to describe the ability of sonication to dislodge biofilm. Our intention was to verify the presence of biofilm by randomly choosing one implant from each animal to SEM.

#### Bacterial strains and inoculum

A suspension of *Staphylococcus epidermidis* ATCC 35984 was prepared, and serially diluted to 10^5^ CFU/mL as described earlier [14]. The bacterial solution was kept on ice until inoculation of the bone graft implant.

#### Steel implants

A total of 28 steel plates, 5 × 5 × 1 mm, AISI 316L (Skala Fabrikk, Terminalen 6, N-7080 Heimdal, Norway), with surface roughness RA 0.06–0.08 µm, were sterilized.

#### Bone xenografts

Corticospongious bone xenografts were obtained from the ribs of Norwegian white sheep (Slaughterhouse Nortura Malvik®, 7550 Hommelvik, Norway) and prepared as earlier described with minor adjustments [19]. Bone grafts were cut into 14 pieces of ~ 15 mm length. A unicortical hole, 2 mm wide, was drilled at the mid part of the graft into the spongiosa for later bacterial inoculation (Fig. 2). A groove of identical size as the steel plates was punched into each end of the bone graft before sterilization. Finaly, the sterilized steel plates were inserted into the groove at each end of the bone graft under sterile conditions.

#### Animal housing and operation

Seven male albino, outbred Wistar rats (HanTac:WH, Taconic, Denmark), 8 weeks old and 378 (355–393)gram of weight were used. The rats were weighed every week throughout the experiment.

Animal housing was performed as earlier described [22].

The operations were performed in a laminar air flow cabinet under standard operation theatre conditions with minor modifications [22]. The bone grafts were inoculated by injecting 10 µl bacterial suspension of *S. epidermidis* into the pre-drilled hole. The suspension was found to contain 520 CFU at the time of preparation. Subsequently, the bone grafts with its implants were implanted into an intramuscular cavity. In the animal acting as negative in vivo control, the bone grafts with its implants were implanted following the same operation procedure but omitting bacterial inoculation. After closing of the incision with sutures the animals were given analgesia (Buprenorphine; Temgesic, Reckitt & Colman), 0.05 mg/kg).

At the end of the experiment the animals were preoperatively prepared as at the primary operation, and additionally shaved on the abdomen. They animals were first placed on their back, washed with 70% alcohol on the abdomen, and a 5 × 5x5 mm biopsy was taken from the spleen by a laparotomy. Subsequently, they were turned over and the old scar on the back was opened. Any local signs of infection were noted. The two bone grafts were retrieved, and one muscle biopsy, 5 × 5x5 mm, taken from each side. All bone grafts containing the steel plates were split longitudinally using a suture holder while still being in the laminar air flow cabinet. Both steel plates and bone grafts were put separately into sterile plastic containers with two ml NaCl and immediately placed on ice for further processing. After surgery the rats were euthanized with pentobarbital (Mebumal 10%, 0.1 mL/100 g) by intravenous injection in the tail.

#### Postoperative processing of biopsies, bone grafts and steel plates

Spleen and muscle biopsies were ground with mortar and pestle in one ml of NaCl and filtered through a Falcon® 70 µm Cell Strainer. Then, 50 µl of the suspension was seeded onto blood agar plates for counting of CFU. Bone grafts without the steel plates were grounded with mortar and pestle in one ml NaCl, and additionally vortex mixed in three ml NaCl for 30 s. They were subsequently sonicated in 10 ml NaCl at 100% effect (800 W) for five minutes at room temperature (see below) before filtering through a Falcon® 70 µm Cell Strainer. Aliquots were obtained for quantitative culture and quantitative PCR. Each steel plate was rinsed three times in separate wells (Nunc 24-well cell culture plate) containing two mL sterile NaCl and gently vortexed at 400 rpm for 10 s. One mL sterile NaCl was gently poured over the plate from a pipette during transfer to the next well to minimize carry-over of planktonic bacteria.

#### Sonication of steel plates

A BactoSonic® sonicator (Bandelin electronic GmbH & Co. KG) was operated according to the manufacturer’s operating instructions and as previously described [14].

The sterile test tube containing one steel plate in 10 mL sterile saline with the surface to be investigated facing upwards were sonicated at 100% effect (800 W) for 5 min at room temperature. The sonicate was then aspirated and transferred to sterile test tubes before quantitative bacterial analyses by culture and qPCR.

#### Quantification of bacteria in rinsing fluid and after sonication

Fifty µL of undiluted and diluted rinsing fluid and sonicate were seeded onto blood agar plates for counting of CFU. Rinsing fluid and sonicate was also quantified by qPCR. Pilot studies showed that staining with LIVE/DEAD ™ BacLight ™ Bacterial Viability Kit did not affect bacterial growth.

#### Quantitative PCR

A real-time PCR was designed after construction of primers and TaqMan probe targeting the *rpoB* gene in *S. epidermidis*. After comparison of available *S. epidermidis* sequences in GenBank the following nucleotide sequences were selected: Forward primer: 5’-TGAAACGCCATATCGTAAAGTG-3’, reverse primer: 5’- CAAGTCTAGAATTAGCCTGT-3’, TaqMan probe: 5’ TAGCTATCCTCTTCATCAGCTGTC-’3 [23]. Lysis of bacteria was performed in TE-buffer with the addition of lysostaphin and Proteinase K. NucliSENS® easyMag (bioMerieux) was employed for DNA extraction and PCR was run on a CFX96 Touch Real-Time PCR Detection System (BIO-RAD). DNA was quantified after lysis of five colonies of *S. epidermidis* ATCC 35984 by Qubit Fluorometric Quantification and dilutions were used to construct a standard curve.

#### Staining and visualization of bacteria with epifluorescence microscopy before and after sonication

Staining of the biofilm was obtained with LIVE/DEAD ™ BacLight ™ Bacterial Viability Kit (Thermo Fischer Scientific, L7012) according to the manufacturer’s protocol. The plates were placed onto the object glass with an integrated coordinate system (Ibidi µ-Slide 8 Well Grid 500, uncoated).

An inverted EVOS™ FL Auto 2 Imaging System enabled visualization of the entire surface. Gain and time of exposure were adjusted to avoid picture saturation and kept constant throughout the experiment. Staining was repeated and imaging performed with identical settings after the sonication procedure to visualize remaining biofilm bacteria.

#### Measurement of area covered by biofilm

Based on pilot studies and preliminary results, it became clear that the previously applied thresholding method for quantifying the area covered by biofilm was inadequate (Sandbakken et al. 2020). Consequently, we developed a new image analysis pipeline capable of differentiating between fluorescent areas and background signals across varying levels of signal intensity in an in vivo biofilm (appendix).

#### Preparation for SEM

The specimens were fixed with a solution of 2.5% glutaraldehyde with 2% paraformaldehyde and 0.075% Ruthenium Red in 0.1 M Hepes buffer for 4 h at room temperature, washed in 0.1 M Hepes buffer and subsequently dehydrated using increasing ethanol concentrations (10, 25, 50, 70, 90, 2 × 100%), for 5–10 min each followed by drying using hexamethyldisiloxane (HMDS) (50% diluted with ethanol once and 100% HMDS twice), for 20 min each and transferred to a desiccator to avoid water contamination. After drying, the samples were mounted on aluminum pin with double sided carbon tape and sputter coated (Leica ACE600) with 30 nm Gold/Palladium. Samples are examined using a scanning electron microscope (VolumeScope SEM, Thermo Fischer Scientific) at a voltage of 10 kV.

#### Statistics

Statistical analyses are performed using the software package IBM SPSS Statistics for Windows, Version 29.0.1.0 (171) Armonk, NY: IBM Corp. Data are presented as median (total range). Correlations between CFU/plate and DNA copies/plate are analyzed with Spearman’s Rho. The three plates from each animal were considered dependent variables. Based on pilot studies, the difference of fluorescent area measured before and after sonication is analyzed with Wilcoxon’s signed rank test by comparing the median percentage value from the three plates at a significance level *p* = 0.05 and 95% confidence intervals. Area covered by biofilm is presented as median (total range) and in boxplots.

#### Confounding variables

Choice of animal strain can be a confounding factor, particularly regarding immune response. We used a common outbred strain with genetic diversity to introduce natural variation. In our model, the bone graft facilitates a chronic infection that resists the host immune response. Further, research animals were allowed to adjust during the acclimatization period to a new environment, reducing stress and stabilizing physiological and behavioral responses before starting the experiments. This adjustment period helps ensure more reliable, reproducible results by minimizing confounding effects caused by environmental stress.

## Results

At the end of the study period all animals were in good health and had gained 74 (71–96) grams of weight. The surgical wounds were healed with no signs of infection. Bone grafts were substantially softened at the time of harvesting and could be separated from the steel plates with a suture holder. In the tissue surrounding the bone grafts there were no macroscopic signs of any infection, that is, no visible purulence and no gangrenous or necrotic tissue. This applied to both infected animals and negative control. All spleen biopsies taken before sacrifice were culture negative.

The results of bacterial analyses are shown in Table 1.

**Table 1:** overall results

Animal No	Plate No	Infected	Muscle biopsy	Bone graft CFU/graft	Rinsing fluid		Sonicate		Fluorescent area		SEM
					CFU/plate	Copies/plate	CFU/plate	Copies/plate	% pre sonication	% post sonication	
1	1	yes	Moderate	4,30E + 07	0	447	100	1601	5.72	0.03	
	2	yes			0	342	*	*	9.7	*	Pos
	3	yes	Sparse	4,50E + 07	460	1453	800	1295	12.9	0.27	
	4	yes			60	478	0	178	2.76	0.06	
2	5	yes	Moderate	1,60E + 07	20	929	100	345	10.15	0.08	
	6	yes			30	627	*	*	15.71	*	Pos
	7	yes	Moderate	2,80E + 07	140	2783	200	1404	5.26	0	
	8	yes			80	499	100	736	19.5	0.05	
3	9	yes	Moderate	2,40E + 07	40	246	1000	204	2.3	0.06	
	10	yes			200	3859	*	*	25.35	*	Pos
	11	yes	Moderate	4,10E + 07	40	204	100	342	5.64	0.84	
	12	yes			180	1053	1200	1427	14.64	2.05	
4	13	yes	Sparse	4,20E + 07	80	220	800	517	11.72	0.02	
	14	yes			60	363	100	433	27.19	0.34	
	15	yes	Moderate	3,10E + 07	140	610	*	*	14.37	*	Pos
	16	yes			20	310	400	539	8.41	0.51	
5	17	yes	Moderate	2,00E + 07	280	2090	*	*	25.86	*	Pos
	18	yes			280	1588	2400	1821	2.24	0.04	
	19	yes	Moderate	4,60E + 07	300	1588	600	208	11.26	0.07	
	20	yes			80	7506	1600	224	18.54	0.27	
6	21	yes	Moderate	3,00E + 07	60	543	600	882	18.71	0.1	
	22	yes			100	593	*	*	13.81	*	Pos
	23	yes	Moderate	3,40E + 07	40	119	0	140	2.23	0.17	
	24	yes			80	1018	100	227	13.33	0.02	
7 negative control	25	no	No	No	0	132	*	*	2.12	*	Neg
	26	no			0	151	0	0	6.08	0.08	
	27	no	No	No	0	0	0	0	9.32	0.16	
	28	no			0	ͳ	0	29	18.73	ͳ	

Table 1 contaning all results from experiments, columns presented from left to right: Data is sorted according to animal number 1–7, number 7 being the negative control. Plate number indicates the row containing all data connected to this specific steel plate implant. Bacterial growth in the muscle tissue surrounding the bone graft was semi-quantitatively assessed as sparse, moderate or abundant growth. Number of bacteria found in the bone graft, rinsing fluid and sonicate is reported as CFU/bone graft and CFU/plate or copies/plate, respectively. Fluorescent area measured as a percentage of the steel plate surface is reported before and after sonication. The last column indicates the 7 plates randomly chosen for SEM to verify biofilm formation

1. * = Plates randomly chosen for SEM

2. ͳ = missing value, plate lost for analysis

All infected bone graft implants displayed abundant growth of *S. epidermidis; 3.25* (1.6–4.6) 10^7^ CFU/graft. Biopsies from the muscles surrounding the bone grafts in the infected animals showed sparse to moderate growth of *S. epidermidis.* In none of the animals we observed any contaminating bacterial growth. No bacterial growth was observed in the negative control animal.

A total of 21 steel plates, of which three were negative controls, were subjected to sonication (Fig. 1). Of 18 steel plates from the six infected animals 16 were sonicate culture positive with 500 (100–2400) CFU/plate. By qPCR *S. epidermidis* was detected from all 18 plates. The number of bacterial DNA-copies dislodged by sonication was low 475 (140–1821) copies/plate whereas the number was somewhat higher in rinsing fluid 602 (119–7387). All qPCR analyses were negative in the negative control animal. A positive correlation between CFU and DNA-copies of *S. epidermidis* was found in both sonicate 0.602 (CI 0.218–0.82) (*p* < 0.004) and rinsing fluid 0.776 (CI 0.56–0.89) (*p* < 0.001).

### Visualization of established biofilm

**Fig. 3:**
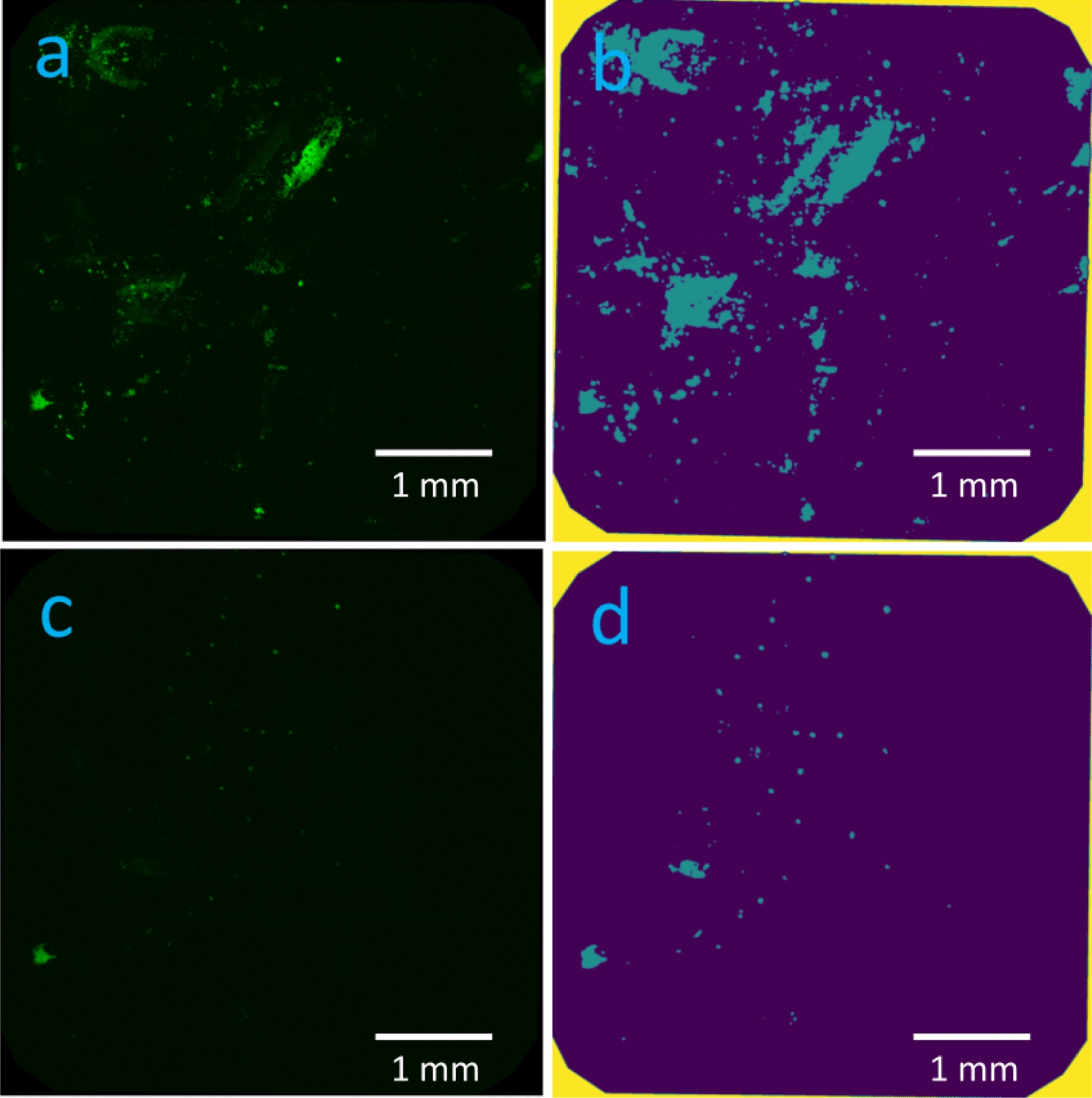
Area of fluorescent material. The figure shows images from one plate before (**a**, **b**) and after sonication (**c**, **d**). Raw images (**a**, **c**) are segmented (**b**, **d**) showing fluorescent material as blue area on purple background signal. The blue area is analyzed as percentage of the plates surface covered with fluorescent material. The margins of the plates are minimally cropped to avoid fluorescent artefacts known as “rand phenomenon”

**Fig. 4:**
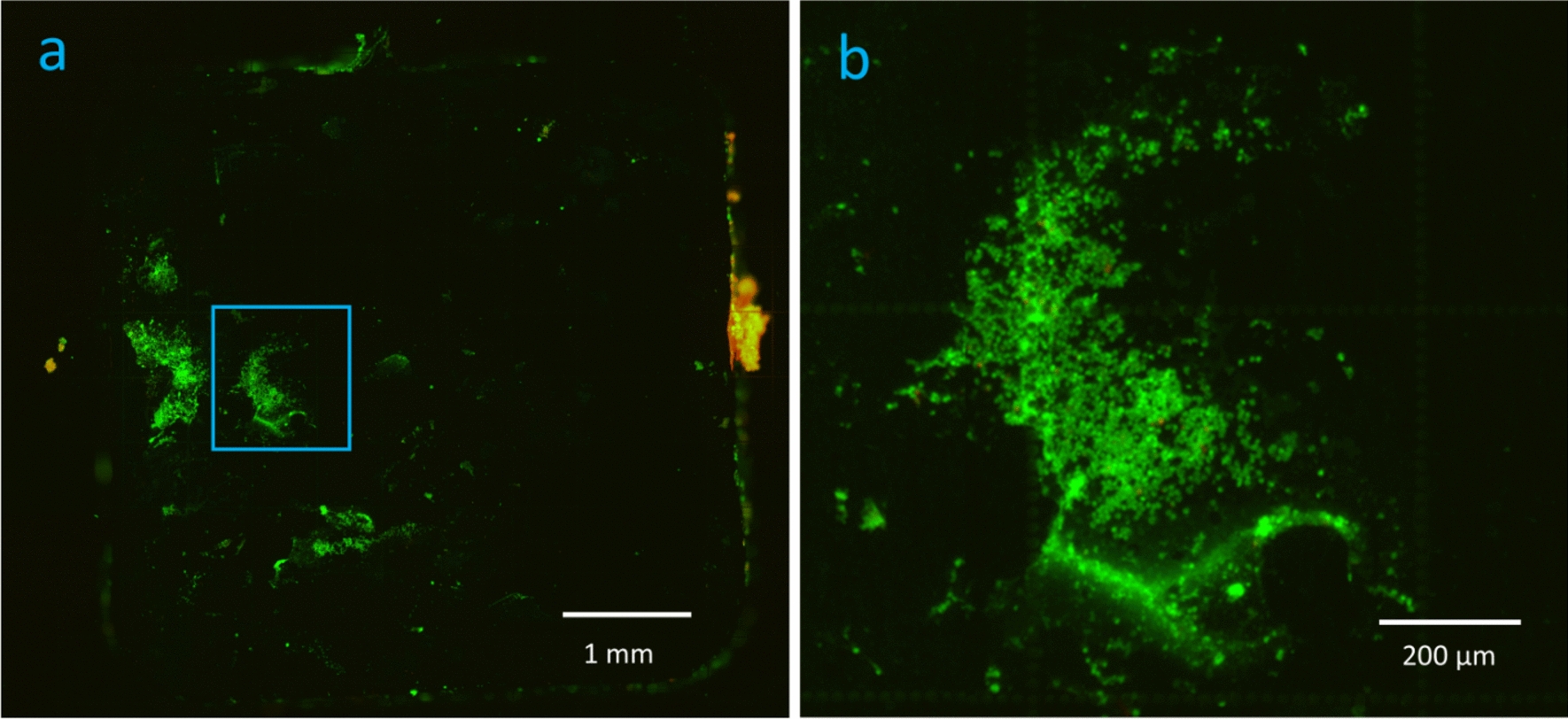
Fluorescense on uninfected plates. Negative control plate (**a**) showing fluorescent material. Section from blue square (**b**) show cells similar to those vizualized on infected plates in Fig. 6, a and c, probably representing host cells

**Fig. 5:**
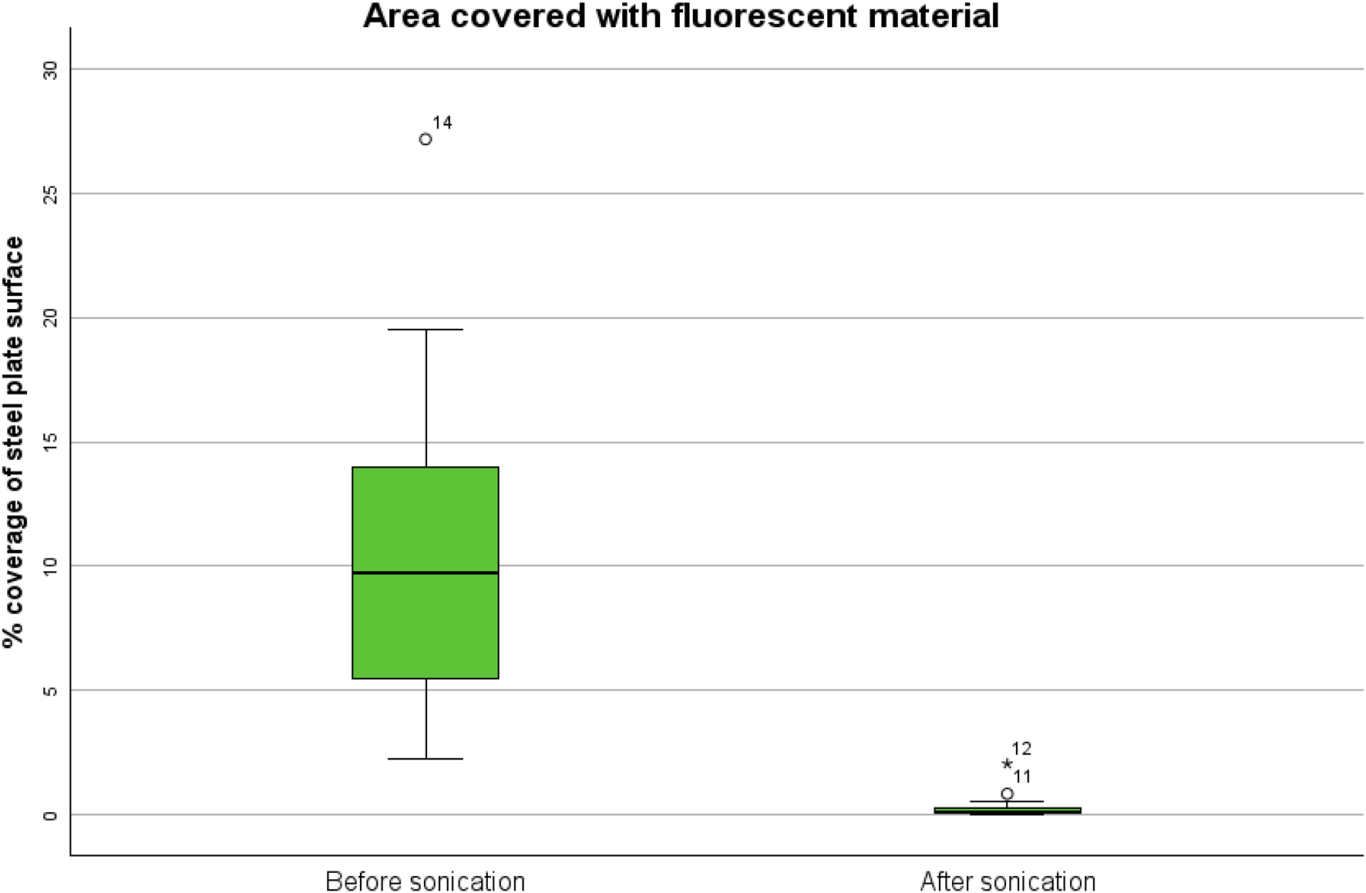
Box plots showing the percentage of the plates covered with fluorescent material before (left) and after sonication (right). Outliar numbers refers to plate number in Table 1

Qualitative evaluation with epifluorescence microscopy of biofilm bacteria before sonication revealed scattered clusters of fluorescent material. We successfully developed an image analysis pipeline capable of differentiating between fluorescent areas and background signals across varying levels of signal intensity in an in vivo biofilm (appendix). The fluorescent areas could be visualized and quantified as a percentage of the steel plate surface (10.2 (5.6–13.3) %, Fig. 3). However, infected plates could not be distinguished from negative controls as similar fluorescent material was present (Fig. 4). Measurement of corresponding plates before and after sonication showed that most of the fluorescent material was dislodged by sonication (0.1 (0.02–0.84) %, Fig. 5). The difference in area covered by fluorescent material before and after sonication was (10.1 (5.7–12.3) %, *p* = 0.018).

**Fig. 6:**
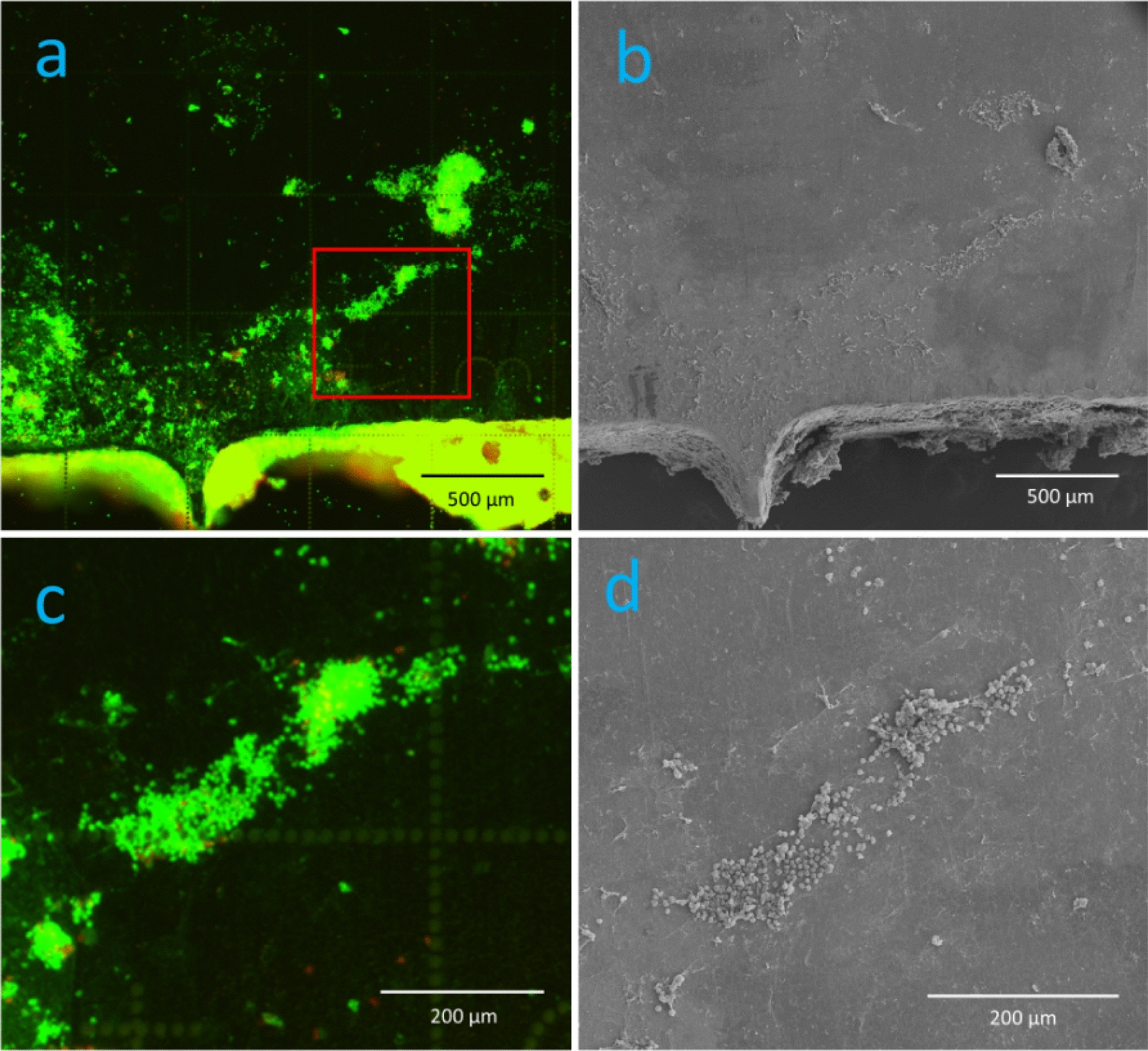
Visualisation of the plate surface before sonication with epifluorescence microscopy and SEM. Epifluorescence microscopy (**a**) revealed circular cells. The relief of the fluorescent material can be recognized with SEM (**b**). The circular cells in the red square (**a**) are visualized at a greater magnification (**c**), showing circular eukaryotic cells both with epiflurorescence microscopy (**c**) and SEM (**d**)

**Fig. 7:**
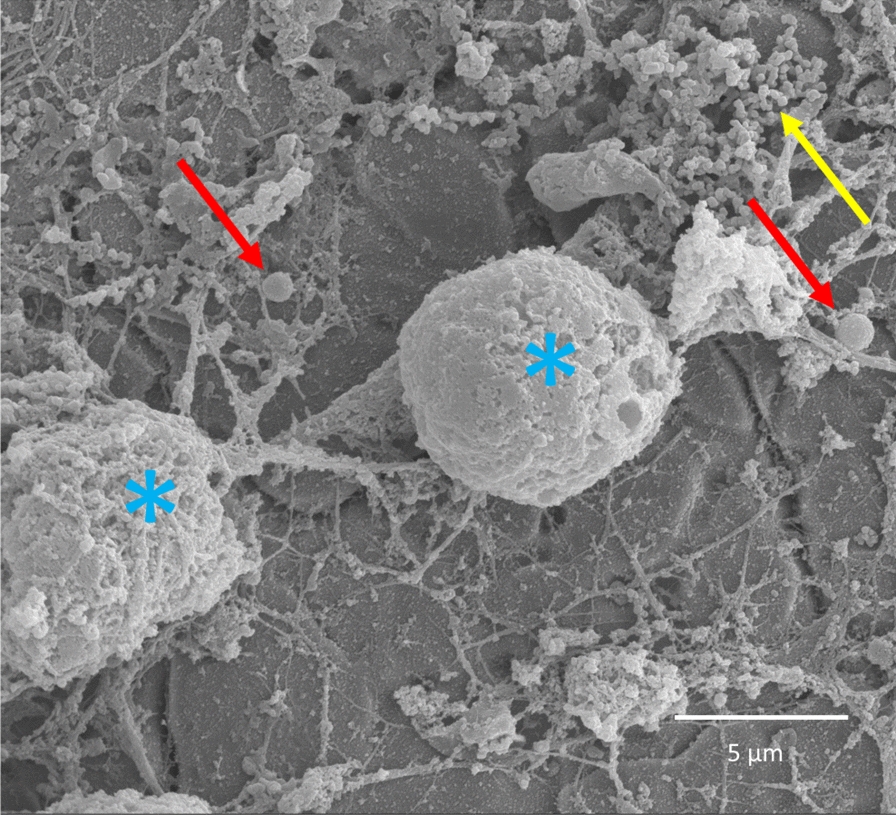
SEM showing eukaryotic cells (blue asterix) clearly distinguishable from coccoid bacteria (red arrow). Small granules are also shown (yellow arrow)

**Fig. 8:**
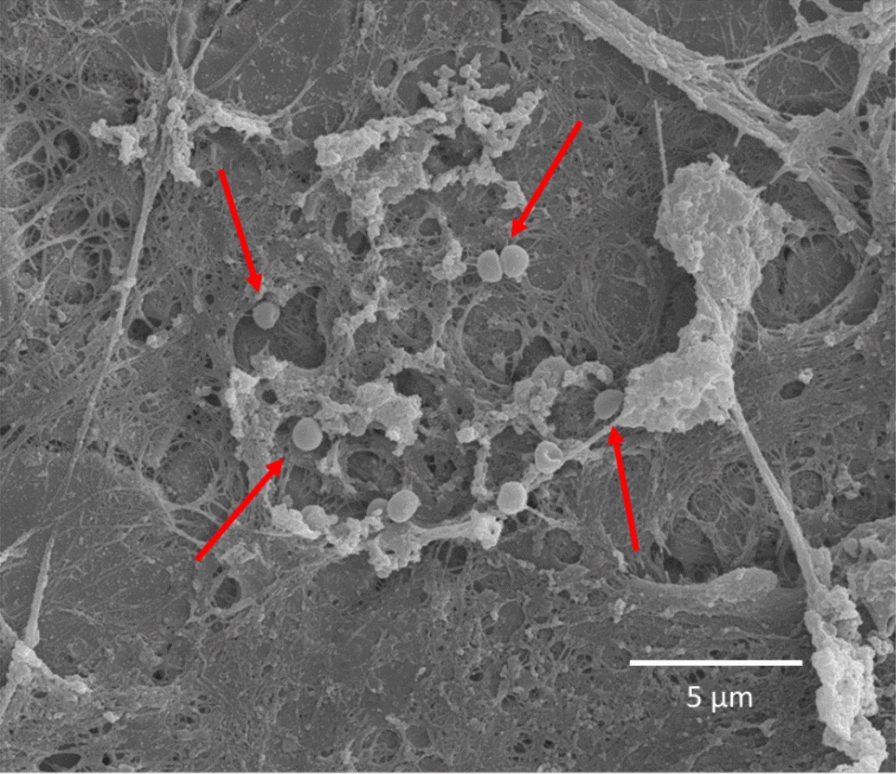
SEM showing small clusters of coccoid bacteria (red arrows)

**Fig. 9:**
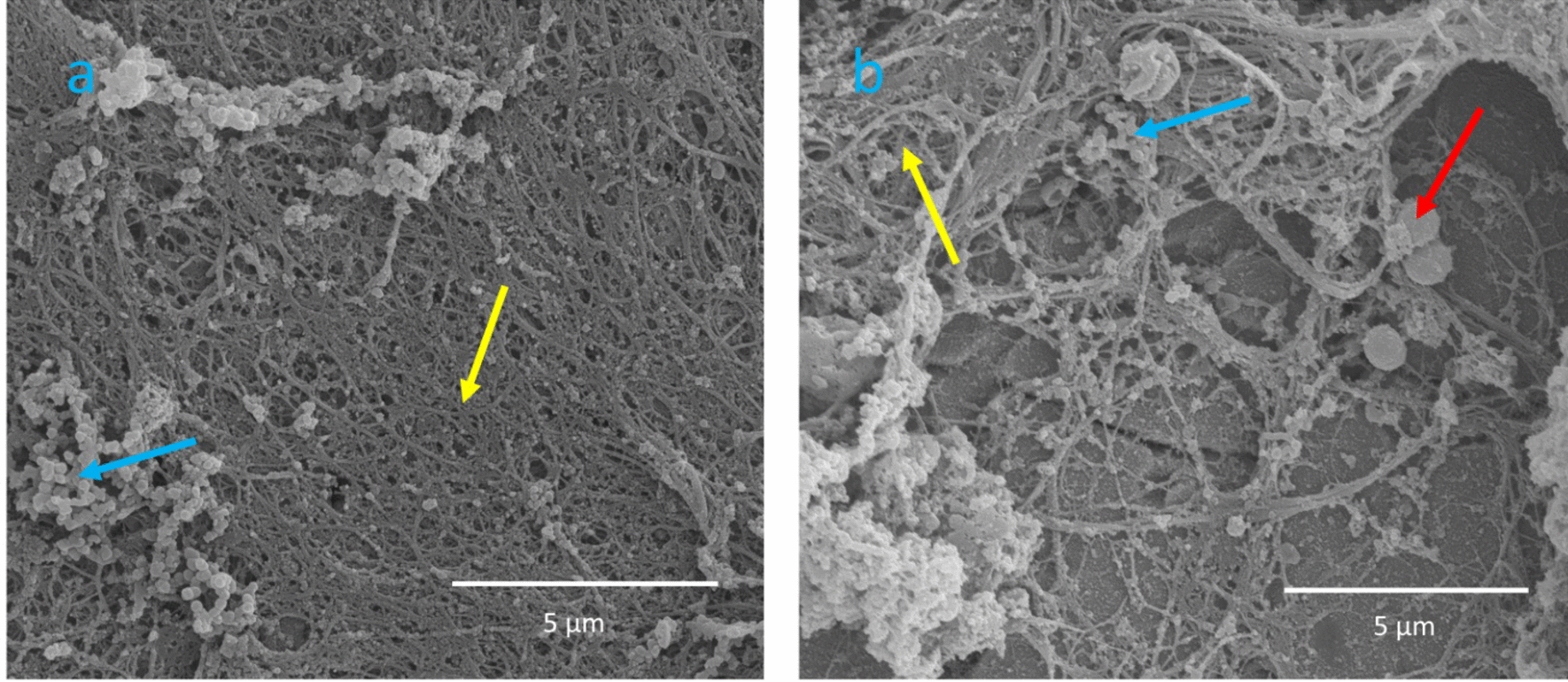
SEM of the negative control plate (**a**) showing matrix-like structures (yellow arrows) and granules (blue arrows). The granules are spherical and appearing in clusters, but clearly smaller and thus distingusihable from coccoid bacteria (red arrows) present on an infected plate (**b**)

Within areas with fluorescent material eukaryotic cells with a macroscopic appearance of circular elements were evident (Fig. 6c, d). Only further investigation with SEM at higher magnification of the same fluorescent material could identify single coccoid bacteria and distinguish them from eukaryotic cells and small granules (Fig. 7). Some smaller colonies of bacteria were found on other plates (Fig. 8). No bacteria were identified with SEM on the negative control plates. Similar matrix-like material and granules less than 1 µm (Figs. 7 and 8) were found on both infected plates and the negative control plate (Fig. 9a).

Based on qualitative observations coccoid cells consistent with *S. epidermidis* could be identified with SEM within corresponding fluorescent areas. By comparing results from epifluorescence and SEM it is evident that the number of bacteria in the biofilm is low, consistent with low numbers of bacteria in cultures and qPCR from sonicate. Taken together we conclude that a biofilm infection, though with a low number of bacteria, was established on all infected plates.

## Discussion

In this study we used a chronic low-inoculum orthopedic implant infection model without local and systemic signs of infection in the animals [15, 19]. To our knowledge no other animal model has shown a consistent establishment of a chronic orthopedic implant infection from such a low inoculum as in the present study. Chronic in vivo biofilm infections with *S. epidermidis* are less described than those with *S. aureus,* and so this study may shed light on the nature of chronic orthopedic implant infections. Further, verification with SEM of bacteria embedded in the biofilm also strengthens this study as few others convincingly present images of *S. epidermidis* on the surface of implants from in vivo model infections. This model represents a chronic orthopedic infection by causing a local low-grade disease with a small inoculum of a low-virulent bacterium. Although a period defining chronicity is not formally defined for in vivo studies, our 20-day observation period aligns with other studies [16, 17, 24, 25]. Additionally, extending this period to 42 days in our previous study yielded comparable bacterial counts after sonication [19].

The number of bacteria dislodged by sonication was surprisingly low despite abundant growth in the bone graft surrounding the steel plates. We consider a biofilm infection was established on all steel plates in the present orthopedic implant infection model based on three observations: The demonstration of a subacute/chronic peri-implant infection, coccoid bacteria visualized by SEM and release of bacteria by sonication. These findings are in accordance with our earlier methodological study, which showed low numbers of bacteria in the sonicate whereas the bone grafts displayed abundant bacterial growth [19]. Interestingly, in a clinical prospective study on chronic PJI with prosthetic loosening, we also found more bacteria in the “interface membrane” compared to the sonicate [10].

In the present in vivo model, a chronic infection was established from a low inoculum. The period during which the biofilm infection has developed is probably an important factor for the number of bacteria embedded in the biofilm. In a clinical study, it is hypothesized, that a longer period is associated with higher numbers of bacteria embedded in biofilm on the implant surface. A shorter period, such as acute infections, are associated with higher numbers of bacteria in the surrounding (interface) tissue [24]. The latter statement is supported by another study emphasizing that high virulence is associated with positive sample tissue culture [2]. In vivo models suggest a period of approximately 14 days as optimal based on observation from bioluminescence and SEM showing peak infection levels after 14 days after inoculation with *S. aureus* [16, 17, 25, 26]. Experimental in vivo studies operate within shorter periods of time than clinical infections based on these observations. Therefore, it is difficult to draw conclusions regarding number of bacteria in biofilm or tissue cultures based on in vivo studies.

Our study showed that the number of bacteria quantified with qPCR correlated with the number of CFU in sonicate, but we could not detect a higher bacterial load with qPCR. It is claimed that PCR is a more sensitive tool than cultures of sonicate [27, 28]. Interestingly, qPCR could not detect a higher bacterial load than culture but was able to detect 18/18 plates as infected whereas cultures of sonicate detected 16/18 plates as infected.

The rinsing procedure of the steel plates before sonication was employed to remove planktonic bacteria in the sonicate. Results (Table 1) showed that the three rinsing steps described was not sufficient to completely remove all planktonic bacteria. In all samples, however, the number of bacteria in the sonicate was higher than in the rinsing fluid measured by culture showing that the rinsing steps removed a considerable number of loosely attached bacteria not embedded in biofilm. qPCR on the other hand, showed much higher numbers of bacteria in the rinsing fluid compared to quantification with culture. Considering that qPCR also measures bacterial DNA from dead bacteria, probably in abundance in the infected xenograft, this finding may not be surprising.

We further successfully employed visualization of fluorescent material on the surface of the steel plates with epifluorescence microscopy which allowed us to compare areas covered with fluorescent material before and after sonication as previously described in vitro [14]. Fluorescent material attached to the surface was completely dislodged by sonication. We were able to accurately distinguish fluorescent areas from background signal. However, it was impossible to distinguish infected plates from negative controls with epifluorescence. Only direct comparison of corresponding fluorescent areas with SEM allowed us to confidently identify areas containing coccoid bacteria and demonstrated that fluorescence originated mainly from eukaryotic cells and small granules.

Furthermore, the misinterpretation of host eukaryotic cells as bacteria was a substantial pitfall in the present study as described earlier [20]. A thorough examination of cell morphology and size is necessary to distinguish eukaryotic from prokaryotic cells [29]. This observation is supported by other studies emphasizing the risk of misinterpretation of fluorescence from hemosiderin granules as evidence for bacterial biofilm [20]. The small bacterial colonies visualized by SEM can be considered as a proof of biofilm establishment, but measurement of area covered by biofilm must be interpreted carefully. Fluorescence does not necessarily represent bacteria as stated above. Measurement of area covered by biofilm with SEM is possible but brings the question whether all material really represents biofilm [16]. Tomizawa et al. report that “host tissue” is evident on the negative control. The same observation was made in our study. Host material on negative controls resembled biofilm matrix seen on infected plates (Fig. 8). The fluorescent material in the negative controls likely comes from host material, making it difficult to distinguish them from infected plates using LIVE/DEAD™ staining. Using a bacterium-specific fluorophore would probably have reduced these artefacts and thus enhance the ability of epifluorescence microscopy to differentiate infected plates from negative controls. Although areas with fluorescent material visualized with epifluorescence correspond to areas on SEM with eukaryotic cells in our study, measurement of fluorescent area does not represent the number of bacteria in the biofilm. In fact, SEM was decisive to distinguish infected plates from negative controls in our study as both infected and negative plates appeared similar with epifluorescence microscopy. One must consider that in vivo biofilm has a heterogenous appearance and that the sparse number of bacteria might be the phenotype of a biofilm established from a low-virulent bacterium as *S. epidermidis* on stainless steel. This appears somewhat in contrast to the well-established mushroom model based on studies of *Pseudomonas aeruginosa* [30, 31].

Only a few in vivo models describe biofilm establishment with low-virulent bacteria, such as *S. epidermidis,* in a chronic orthopedic implant infection model [16, 19, 32–37]. In three of these studies, which included positive and negative controls, SEM verified biofilm formation on the implant [16, 19, 35]. As in the present study, relatively low numbers of bacteria were observed. A fourth study also presented biofilm with SEM on a polyethylene washer [37]. However, it is not obvious on the presented images in two of these studies [35, 37] that the cells pictured as biofilm-embedded coccoid cells are in fact bacteria (~ 1 µm) and not eukaryotic host cells.

Relatively low numbers of bacteria attached to the implants is in accordance with our results. In studies using sonication as a method for biofilm quantification the amount of bacteria from the implant was in the range 10^2^ CFU/mL [19], 10^2^ CFU/implant [16], 10^5^ CFU/wire [36] and 18–268 CFU/implant cm^2^ [33]. Direct comparison between these studies is somehow problematic due to methodological differences, e.g. lack of rinsing procedure prior to sonication [33, 36]. Period of biofilm development also differ substantially among the studies. Interestingly, poor attachment of *S. epidermidis* to steel is reported by two in vivo studies independently [19, 33] and further described for *Cutibacterium acnes* where biofilm development is dependent on surface qualities of the implant [38].

Only few studies of *S. epidermidis* report convincing verification of biofilm-embedded bacteria attached to the surface of the implants [16, 19]. Verification of *S. aureus* biofilm with SEM is described [17, 26, 39–42]. Two of these studies distinguish the morphology of coccoid bacteria from eukaryotic cells convincingly [17, 26], whereas the true value of SEM as a tool to verify biofilm-embedded bacteria is not fully utilized due to lower resolution and magnification in the other studies we are aware of [39–42]. The high virulence of *S. aureus* compared to *S. epidermidis* as well as methodological differences make direct comparison between in vivo studies difficult. Generally, in vivo models with *S. aureus* are in a vast majority but seem less relevant for understanding *S. epidermidis *in vivo biofilm development. However, the natural history of biofilm development is nicely described for *S. aureus* [17] and should be done likewise for *S. epidermidis.*

The sonication procedure was carried out according to clinical practice [6] and was able to dislodge all fluorescent material from the surface of the steel plates. We did not see a variable effect of sonication as reported earlier from in vitro studies [14]. Microscopic results show that bacteria seem to be a minor component of the biofilm in this in vivo model, and one must consider that in vivo biofilm responds differently to sonication than in vitro biofilm.

The diagnostic value of sonication as a method to detect implant infections was similar (16/18) to sensitivity reported in clinical studies [2, 10]. Detection with qPCR could detect all infected plates (18/18).

We are aware of certain weaknesses of this study. The application of a xenograft sustaining a chronic infection and possibly promoting influx of inflammatory cells does not necessarily resemble a chronic implant infection. Further, it is limited to studying a low-virulent bacterial species such as *S. epidermidis*, and only employing stainless steel as substratum for biofilm generation. Although the reference strain (ATCC35984) is shown to be a biofilm forming strain [43] it is not certain that the in vivo phenotype shares the same characteristics when it comes to biofilm formation. Clinical strains from patients with biofilm related PJI might be equally relevant for in vivo studies.

Randomization was only done when selecting plates for SEM. Randomization and blinding of infected animals and control animals could have been done. Blinding of the information infected/not infected plate when interpreting the epifluorescence and SEM-pictures would have added integrity to the results.

The sample size was based on earlier studies [14, 44] and could have been larger to enhance statistical certainty. The number of animals used was pragmatically balanced with the workload manageable by our personnel and aligned with animal welfare requirements.

Though epifluorescence microscopy with LIVE/DEAD™ staining is widely used and allows morphological cell distinction, epifluorescence microscopy is not specific to microorganisms. The lack of specificity in vivo requires alternative fluorescent methods to reliably identify the bacteria. Using a Green Fluorescent Protein (GFP)-expressing *S. epidermidis* strain could be such a method [45].

## Conclusions

In conclusion, we established a chronic low-grade orthopedic implant infection model without general infection signs. This was supported by culture, qPCR, and biofilm analysis with epifluorescence and SEM. The low inoculum, compared to other models, enhances its relevance for chronic PJIs. Sonication effectively dislodged biofilm bacteria, with qPCR yielding positive results in 18/18 plates versus 16/18 for sonicate cultures. Sonication did not show variable effectiveness, indicating it is a valuable addition to, but not a replacement for biopsy cultures in cases of implant-associated infections with low-virulence microorganisms.

## Data Availability

The datasets used and/or analyzed during the current study are available from the corresponding author on reasonable request. The code and raw data required to reproduce the image analysis pipeline results are available at (https://github.com/adiezsanchez/biofilm_meas) [46].

## Appendix

Before training the classifier and analyzing the images, the margins of the plates were manually cropped using a predefined shape to eliminate confounding fluorescence artifacts. The classifier was trained on manually annotated images to distinguish between biofilm and background. The analysis pipeline employs random forest classifiers implemented using scikit-learn, with subsequent GPU acceleration achieved by transforming the trained classifier into OpenCL format. This approach leverages GPU acceleration to classify pixels as either biofilm or background using the pyclesperanto-prototype library. The entire process is packaged within the APOC (Accelerated Pixel and Object Classifier) repository (https://github.com/haesleinhuepf/apoc). This methodology allows for efficient and accurate quantification of the area covered by fluorescent material, expressed as a percentage of the total plate surface.

The code and raw data required to reproduce the analysis pipeline results are available at (https://github.com/adiezsanchez/biofilm_meas) [46].

## Abbreviations

SEM: Scanning electron microscopy
ATCC: American type culture collection
qPCR: Quantitative real-time PCR
CFU: Colony forming units
PJI: Prosthetic joint infection
EBJIS: The European bone and joint infection society
MSIS: The muskuloskeletal infection society
GFP: Green fluorescent protein
